# Supplementary material for: A New Technique for Determining Micronutrient Nutritional Quality in Fruits and Vegetables Based on the Entropy Weight Method and Fuzzy Recognition Method
Source: Foods. 2022 Nov 28;11(23):3844. doi: 10.3390/foods11233844 (PMC9740144; doi:10.3390/foods11233844)
Supplement: Supplementary file 1 [file foods-11-03844-s001.zip › foods-1909321-supplementary.pdf]

Table S1. The proportion of the population whose daily vitamin intake was lower than the RNI between 2010 to 2013 (%). The intake of vitamin E uses AI. The data source population represents the reference intake of dietary nutrients for people aged 18-50.

	Vitamin A	Vitamin B1	Vitamin B2	Niacin	Vitamin C	Vitamin E
Average	95.2	87.3	96.4	26.3	76.6	/
City	94.5	90.6	93.3	22.5	75.4	/
Rural	95.9	84.1	95.8	28.1	77.8	/
Average intake of school-aged children	86.0	87.0	93.2	/	83.6	/
Average intake of urban school-aged children	80.4	88.4	88.8	/	83.8	/
Average intake of rural school-aged children	89.8	86.1	96.2	/	86.8	/
Average intake of the elderly	88.8	93.08	96.5	/	80.3	23.9
Average intake of urban elderly	85.5	93.8	94.9	/	77.1	20.3
Average intake of rural elderly	92.9	92.3	98.5	/	84.1	28.3
The main source of food	Vegetables	Noodles	Vegetables	Rice and Meat	Vegetables	Edible Oil and Vegetables

Table S2. The proportion of the population whose average daily mineral intake is lower than the RNI from 2010 to 2013 (%). The selenium intake is expressed in μg, while the recommended potassium intake value is expressed as AI.

	Calcium	Magnesium	Potassium	Phosphorus	Iron	Zinc	Selenium
Average	98.5	76.5	83.4	29.6	31.1	53.4	82.2
City	97.9	79.9	/	31.4	31.4	56.5	80.4
Rural	99.0	73.1	/	27.9	30.9	50.4	83.9
Average intake of school-age children	99.6	/	/	/	43.2	53.1	/
Average intake of urban school-age children	99.2	/	/	/	43.0	49.7	/
Average intake of rural school-age children	99.9	/	/	/	46.8	53.8	/
Average intake of the elderly	98.8	/	86.3	/	21.6	66.9	/
Average intake of urban elderly	98.2	/	83.4	/	20.3	66.8	/
Average intake of rural elderly	99.6	/	89.9	/	23.3	67.1	/
The main source of food	Vegetables	Noodles, Rice and Vegetables	Vegetables	Noodles and Rice	Noodles, Rice and Vegetables	Rice	Vegetables

Table S3. The vitamin and mineral content of 20 fruits

Fruits	Vitamins(mg/100g)						Minerals（mg/100g）							
	Vitamin（μgRAE）	Vitamin B3	Vitamin C	Vitamin E (mg/α-TE/d)	Thiamine（B1）	Riboflavin（B2）	Calcium	Potassium	Magnesium	Iron	Zinc	Copper	Manganese	Selenium（μg）
Apples	4	0.2	3	0.43	0.02	0.02	4	83	4	0.3	0.04	0.07	0.03	0.1
Pears	2	0.2	5	0.46	0.03	0.03	7	85	8	0.4	0.1	0.1	0.06	0.29
Peaches	2	0.3	10	0.71	0.01	0.02	6	127	8	0.3	0.14	0.06	0.07	0.47
Chinese Dates	20	0.9	243	0.78	0.06	0.09	22	375	25	1.2	1.52	0.06	0.32	0.8
Apricots	38	0.6	4	0.95	0.02	0.03	14	226	11	0.6	0.2	0.11	0.06	0.2

Cherries	18	0.6	10	2.22	0.02	0.02	11	232	12	0.4	0.23	0.21	0.1	0.07
Grapes	3	0.25	4	0.86	0.03	0.02	9	127	7	0.4	0.16	0.18	0.04	0.11
Pomegranates	5	0.3	8	3.72	0.05	0.03	6	231	16	0.2	0.79	0.15	0.17	/
Blackcurrants	9	0.3	181	1.45	0.05	0.05	55	322	24	1.5	0.27	0.09	0.26	/
Kiwis	11	0.3	62	2.43	0.05	0.02	27	144	12	1.2	0.57	0.87	0.73	0.28
Strawberries	3	0.3	47	0.71	0.02	0.03	18	131	12	1.8	0.14	0.04	0.49	0.7
Oranges	13	0.3	33	0.56	0.05	0.04	20	159	14	0.4	0.14	0.03	0.05	0.31
Kumquats	31	0.3	35	1.22	0.04	0.03	56	144	20	1	0.21	0.07	0.25	0.62
Lemons	3	0.6	22	1.14	0.05	0.02	101	209	37	0.8	0.65	0.14	0.05	0.5
Pineapples	2	0.2	18	0.1	0.04	0.02	12	113	8	0.6	0.14	0.07	1.04	0.24
Bananas	5	0.7	8	0.24	0.02	0.04	7	256	43	0.4	0.18	0.14	0.65	0.87
Lychees	1	1.1	41	0.07	0.1	0.04	2	151	12	0.4	0.17	0.16	0.09	0.14
Mangoes	75	0.3	23	1.21	0.01	0.04	7	153	10	0.5	0.14	0.1	0.24	0.25
Watermelons	14	0.3	5.7	0.11	0.02	0.04	7	97	14	0.4	0.09	0.03	0.03	0.09
Cantaloupes	77	0.73	12	0.05	0.04	0.01	4	190	19	/	0.13	0.01	0.01	1.1

Table S4. The vitamin and mineral content of 24 vegetables.

Vegetables	Vitamins(mg/100g)						Minerals（mg/100g）							
	Vitamin（μgRAE）	Vitamin B3	Vitamin C	Vitamin E (mg/α-TE/d)	Thiamine（B1）	Riboflavin（B2）	Calcium	Potassium	Magnesium	Iron	Zinc	Copper	Manganese	Selenium（μg）
White Radish	2	0.14	19	0	0.02	0.01	47	167	12	0.2	0.14	0.01	0.05	0.12
Carrots	344	0.6	13	0.41	0.04	0.03	32	190	14	1	0.23	0.08	0.24	0.63
Chinese Celery	2	1.26	1	0.08	0.04	0.07	79	441	33	0.5	0.58	0.23	0.33	1.03
Peas	18	2.3	14	1.21	0.43	0.09	21	332	43	1.7	1.29	0.22	0.65	1.74
Lentils	5	0.24	2	0.24	0.05	0.06	54	163	31	0.5	0.26	0.05	0.13	/
Kidney Beans	8	0.26	6	1.24	0.02	0.05	43	196	27	0.6	0.33	0.05	0.26	0.04
Eggplant	4	0.6	5	1.13	0.02	0.04	24	142	13	0.5	0.23	0.1	0.13	0.48
Tomatoes	31	0.49	14	0.42	0.02	0.01	31	197	12	0.4	0.24	0.05	0.06	0.5
Peppers	116	0.8	144	0.44	0.03	0.06	9	278	18	0.5	0.18	0.06	0.09	0.12
Cucumbers	8	0.2	9	0.49	0.02	0.03	24	102	15	0.5	0.18	0.05	0.06	0.38
Pumpkins	74	0.4	8	0.36	0.03	0.04	16	145	8	0.4	0.14	0.03	0.08	0.46
Chinese Cabbage	7	0.65	37.5	0.36	0.05	0.04	57	137	12	0.8	0.46	0.06	0.19	0.57
Wuta-Tasi	84	1.1	45	1.16	0.06	0.11	186	154	24	3	0.7	0.13	0.36	0.5
Rape	122	0.7	24	0.94	0.01	0.1	153	157	27	3.9	0.87	/	0.13	/

Collard Greens	364	1.09	63	1.12	0.07	0.18	66	395	53	1.6	0.56	0.06	0.4	/
Broccoli	13	0.73	56	0.76	0.06	0.08	50	197	22	0.9	0.46	0.03	0.16	0.43
Spinach	243	0.6	32	1.74	0.04	0.11	66	311	58	2.9	0.85	0.97	0.1	0.66
Celery	2	0.22	4	0.2	0.01	0.03	36	15	15	0.2	0.1	0.02	0.06	0.1
Lettuce	2	0.52	6	0.08	0.02	0.01	14	91	7	0.2	0.12	0.01	0.06	0.04
Red Amaranth	124	0.6	30	1.54	0.03	0.1	178	340	38	2.9	0.7	0.07	0.35	0.09
Mustard Greens	142	0.5	72	0.64	0.02	0.11	28	224	18	1	0.41	0.1	0.7	0.53
Yam	3	0.3	5	0.24	0.05	0.02	16	213	20	0.3	0.27	0.24	0.12	0.55
Taro	1	0.28	1.5	0	0.05	0.02	11	25	19	0.3	0.19	0.06	0.3	0.91
Potatoes	1	1.1	14	0.34	0.1	0.02	7	347	24	0.4	0.3	0.09	0.1	0.47

Table S5. The Vitamin Index and Mineral Index results in the fruits.

	Vitamin Index				Mineral Index			
	Vitamin A Index	Comprehensive Index	Matching Index	Vitamin Index	Calcium Index	Comprehensive Index	Matching Index	Mineral Index
Apples	0.50	0.03	0.55	1.08	0.25	0.21	0.44	0.90
Pears	0.25	0.03	0.57	0.85	0.5	0.24	0.47	1.20
Peaches	0.25	0.05	0.60	0.90	0.25	0.32	0.48	1.05
Chinese Dates	1.00	1.00	0.62	2.62	1	0.98	0.65	2.63
Apricots	1.00	0.14	0.63	1.76	0.7	0.58	0.52	1.80
Cherries	0.75	0.10	0.67	1.52	0.75	0.58	0.54	1.87
Grapes	0.25	0.03	0.58	0.86	0.4	0.33	0.49	1.23
Pomegranates	0.50	0.05	0.66	1.22	0.25	0.57	0.58	1.40
Blackcurrants	0.75	0.73	0.60	2.08	1	1.00	0.58	2.58
Kiwis	0.75	0.28	0.64	1.67	0	0.47	0.62	1.09
Strawberries	0.25	0.19	0.60	1.05	0.75	0.40	0.51	1.65
Oranges	0.75	0.17	0.63	1.55	0.75	0.47	0.50	1.71
Kumquats	1.00	0.23	0.64	1.88	1	0.61	0.54	2.15
Lemons	0.25	0.10	0.68	1.03	1	0.98	0.63	2.61
Pineapples	0.25	0.08	0.61	0.93	0.75	0.32	0.51	1.58
Bananas	0.50	0.05	0.63	1.18	0.5	0.69	0.61	1.79
Lychees	0.25	0.16	0.63	1.05	0.25	0.37	0.51	1.13
Mangoes	1.00	0.32	0.67	1.99	0.5	0.39	0.50	1.39
Watermelons	0.75	0.07	0.58	1.40	0.5	0.27	0.47	1.24
Cantaloupes	1.00	0.29	0.66	1.94	0.25	0.48	0.51	1.24
**Average**	**1.43**				**1.61**			
